# An update on the distribution of *Blastocystis* subtypes in the Americas

**DOI:** 10.1016/j.heliyon.2022.e12592

**Published:** 2022-12-24

**Authors:** Paula Jiménez, Marina Muñoz, Juan David Ramírez

**Affiliations:** a Centro de Investigaciones en Microbiología y Biotecnología-UR (CIMBIUR), Facultad de Ciencias Naturales, Universidad del Rosario, Bogotá, Colombia; b Molecular Microbiology Laboratory, Department of Pathology, Molecular and Cell-based Medicine, Icahn School of Medicine at Mount Sinai, New York City, NY, USA

**Keywords:** *Blastocystis*, STs, Subtypes, Americas, Geographic distribution, Epidemiology, Diversity

## Abstract

*Blastocystis* is an intestinal protist that presents worldwide distribution, colonizes animal and human hosts, and is classified into at least 34 ribosomal subtypes (STs). Herein, we conducted an update based on studies reporting *Blastocystis*-positive samples obtained from diverse hosts in the Americas. We described the distribution throughout the continent by assembling maps representing the distribution of STs and the most important 18S-rRNA alleles. Thirty-nine articles from the previous study, “A summary of *Blastocystis* subtypes in North and South America,” and forty-one additional articles from March 2019 to March 2022 were considered. The most common subtype described was ST3, representing the highest percentage of positive samples. Other recently identified STs include ST12, ST13, and ST16 in humans, and ST10, ST14, and ST17 in animals. Novel subtypes have also been described in this continent. We assembled and updated the distribution of *Blastocystis* in the Americas. We hope this delivers new understandings and knowledge of this microorganism’s prevalence and genetic diversity.

## Highlights

• We identified that thirteen countries in the Americas had reported the presence of *Blastocystis* in the last two decades.
• Not all countries have performed subtyping of *Blastocystis*, and these studies are limited to its identification.
• Colombia has a tremendous variety of subtypes, reporting 18 different subtypes.
• ST1 is the most widely distributed subtype in the Americas.
• The most common subtype is ST3, representing the highest number of reported positive samples in human and animal hosts.

## 1 Introduction

*Blastocystis* is a stramenopile known as the most common enteric protist in humans and animals worldwide [1, 2]. Likewise, its extensive distribution in developed and developing countries has been described [3, 4]. It colonizes multiple host digestive systems, and the parasite transmits through the fecal-oral route, through water, foods, and some animals are considered the most probable means of spread [4, 5, 6, 7, 8]. Due to its high frequency in different species of animals, the hypothesis of zoonotic transmission of *Blastocystis* between animals and humans has recently been recognized [2, 5, 9, 10, 11, 12, 13, 14, 15]. Although, its zoonotic potential and transmission should be considered in further studies [16].

*Blastocystis* is known to cause gastrointestinal infection in humans, but it is not very well known whether *Blastocystis* infection causes gastrointestinal symptoms in animals. However, *Blastocystis* infection has been associated with gastrointestinal illnesses, urticaria, and other extraintestinal signs of the inflammatory response [9, 17, 18, 19, 20, 21]. It has also been reported in asymptomatic patients [22, 23, 24]. Indeed, the relationship between disease and colonization remains unclear. The high prevalence of polyparasitism in the Americas accompanied by nonspecific symptoms does not allow associations between clinical manifestations and the presence of the microorganism or even link it to any ribosomal subtype.

This eukaryotic microorganism has been recognized as an essential component of the gut microbiota. It is suggested that *Blastocystis* role could be related to gut homeostasis by maintaining a higher gut bacterial diversity [3, 20, 25, 26, 27, 28]. Other reports revealed that *Blastocystis* could be a healthy member of the gut microbiota, and its interaction with other microbial communities might regulate the host immune responses [22, 29, 30, 31, 32, 33, 34]. Regardless of studies reporting a decrease in the abundance of beneficial bacterial communities in the gastrointestinal tract or intestinal dysbiosis due to the presence of *Blastocystis* [22, 23, 30, 34, 35].

Currently, there are known 28 *Blastocystis* Subtypes (STs) whose assignment is based on sequence analysis of the small subunit of ribosomal RNA (rRNA-18S) gene and phylogeny using a numbering system based on publication date [16]. Various tools have been used for its subtyping; these tools include partial amplification of the 18S-rRNA gene, a barcoding method that uses specific primers from a 600 bp region of length [36, 37, 38], and next-generation amplification sequencing by Illumina targeting a variable region of the equal length [39, 40]. Also, recent approaches have focused on third-generation sequencing using the MinION by Oxford Nanopore Technologies, the first tool that uses nanopore machinery to obtain complete sequences (1800 pb) from the *Blastocystis* 18S-rRNA gene [16, 41, 42, 43].

In recent years, *Blastocystis* subtyping has evolved to obtain complete sequences of the 18S-rRNA gene, allowing a robust classification to categorize new subtypes, generate reference sequences, and build accurate phylogenetic lineages [16]. Furthermore, these molecular methods have demonstrated a considerable genetic diversity among *Blastocystis* inter and intra-subtype levels; some studies reported that comparing sequences in different hosts could show similar or highly different strains in some STs. Within the described STs, some have been linked to specific hosts. Of the 28 STs reported worldwide, twelve subtypes (ST1-ST10, ST12, ST13, ST14, ST16, and ST23) have been detected in humans and animals [44, 45], ST1-ST4 the most frequent in human samples [46, 47]. Mixed infections have been described in humans and animals, and multiple subtypes have been described within one sample [46, 48, 49, 50].

The distribution of *Blastocystis* in the Americas is not yet fully clarified. However, in the last five years, there has been an increased interest in detecting and identifying *Blastocystis* STs in these countries, significantly impacting this protozoan’s epidemiological and molecular characterization. Environmental aspects in the American continent, such as poverty, sanitation problems, poor access to potable water, internal civil conflicts, and high biodiversity, lead to a high prevalence of this intestinal microorganism which cannot yet be linked to intestinal manifestations, pathogenic potential, or beneficial influence on gut microbiota. Despite the progress, the epidemiological data obtained have not yet been consolidated. Therefore, this study aims to update the distribution of *Blastocystis* throughout the Americas from samples identified in humans and animals. Maps and graphs were constructed to identify the most frequent subtypes and alleles, including an ample discussion of the subtyping methods used.

## 2 Materials and methods

### 2.1 Identifying available data

Considering our previous review, “A summary of *Blastocystis* subtypes in North and South America” [46], and conducting a new literature search by using databases such as PubMed, Science Direct, Scopus, and the Integrated Search System of Universidad del Rosario, Colombia, led us to find forty-one (41) additional articles from March 2019 to March 2022. In total, eighty (80) papers met the requirements to be considered for the development of this study. The keywords included *Blastocystis*, subtypes, STs, molecular characterization, epidemiology, distribution, alleles, genetic diversity, America, intestinal protozoa, and isolates.

This research was geographically limited to the American continent, including three subcontinents: North America, Central America, South America, and thirty-five nations. There were excluded those reports whose samples were taken outside the continent. We incorporated studies from different languages: English, Spanish, and Portuguese. Information was extracted from the articles containing the date of publication, country, and geographic location of sampling. Based on the information from those reports, we established the following inclusion criteria: methods of identification either by microscopy or molecular methods, subtyping including the use of Next-Generation sequencing, and the host from which the samples were obtained.

### 2.2 Data arrangement

Upholding the data extraction methodology of the previous paper, the information and data from each study were collected, including country, samples' exact location, methods of identification and subtyping, number of positive samples, host, subtype, identified alleles, authors (last name of the first author) and year of publication. The data extraction was performed in January and March of 2021 and 2022. The database of the previous study was used to update it with the latest information and complement the prior publication. The current information obtained from those studies which met the inclusion criteria was added to the variables. The samples' geographic location was also paired with their corresponding coordinates (latitude and longitude) from the collected places.

Using the arranged data, maps and graphics were constructed using R programming. Updating the distribution of *Blastocystis* STs throughout the American continent, including specific geographic hotspots of ST’s occurrence in thirteen different countries, revealing the emergence of new subtypes and new locations of identification of this parasite. First, we built a map that contains all subtypes identified in the Americas, in both human and animal hosts. Subsequently, maps were constructed for the most frequent subtypes (ST1, ST2, ST3) and individually containing percentages of each subtype by country. This information was used to build graphs representing the number of alleles and frequency of *Blastocystis* subtypes in the Americas.

### 2.3 Data analysis

Chi-square tests were applied to identify possible associations between variables of interest (STs, hosts, and countries). Multiple comparisons between different categories were made by implementing post hoc tests through the chisq.posthoc.test function included in the vcd package R software implements pairwise comparisons using Bonferroni as an adjustment method. All statistical analyses were performed using the R software (RStudio Team 2019). All tests of significance were two-tailed, and P-values < 0.05 were considered statistically significant.

## 3 Results

### 3.1 Frequency of *Blastocystis* in the Americas

**Figure 1:**
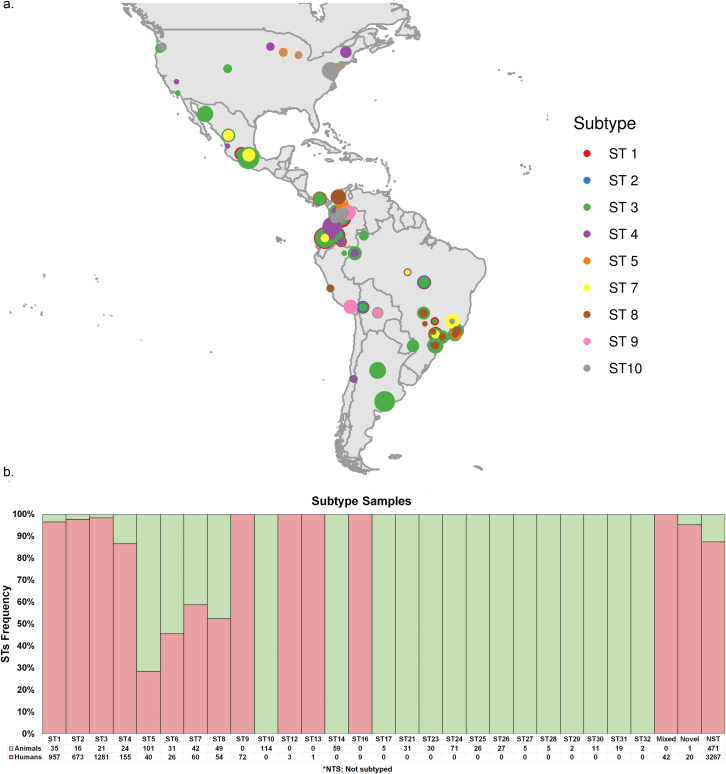
Distribution of *Blastocystis* subtypes in North and South America based on the positive samples reported by country and hosts. a. Distribution of the most frequent *Blastocystis* subtypes, each subtype is separated by color, showing the sample size per subtype for each country represented in circles. b. Frequency of *Blastocystis* subtypes samples by hosts.

From the eighty articles, which met the selection criteria, it was identified that thirteen countries had reported the presence of *Blastocystis* in the last two decades. These countries are Argentina [24, 47, 51], Bolivia [47, 52, 53, 54], Brazil [39, 47, 55, 56, 57, 58, 59, 60, 61, 62, 63, 64, 65, 66, 67, 68, 69, 70, 71, 72, 73], Chile [74, 75], Colombia [9, 31, 43, 47, 76, 77, 78, 79, 80, 81, 82, 83, 84, 85, 86], Ecuador [47, 87, 88], Honduras [89], Mexico [33, 40, 49, 90, 91, 92, 93, 94, 95, 96, 97], Nicaragua [98], Panamá [99], Perú [47, 50, 100, 101, 102, 103, 104], United States [10, 48, 105, 106, 107, 108, 109, 110, 111, 112, 113] and Venezuela [114, 115] (Figure 1a).

The most frequent subtypes found in the Americas were ST3, ST1, and ST2 in both animal and human hosts, ST3 1312/7898 (16,61%), ST1 1002/7898 (12,69%), and ST2 699/7898 (8,85%) respectively, and not subtyped samples 3738/7898 (47,33%). We observed a significant association in human positive samples and ST1 (*p =* 0.014211), ST2 (*p =* 1.00E-06), ST3 (*p =* 5.90E-05) measured by post hoc tests. The number of samples and percentages from other STs (Table 1) (Figure 1 b). It is worth highlighting that not all countries have performed subtyping of *Blastocystis*; these studies are limited to its identification.

**Table 1:** Occurrence of *Blastocystis* in humans and animals from various locations in the Americas.

Subtypes	Number of positive samples	Humans	Animals
*ST1*	1002 (12,69%)	957 (14,37%)	35 (2,92%)
*ST2*	699 (8,85%)	673 (10,11%)	16 (1,34%)
*ST3*	1312 (16,61%)	1281 (19,23%)	21 (1,75%)
*ST4*	179 (2,27%)	155 (2,33%)	24 (2,00%)
*ST5*	141 (1,79%)	40 (0,60%)	101 (8,43%)
*ST6*	57 (0,72%)	26 (0,39%)	31 (2,59%)
*ST7*	102 (1,29%)	60 (0,90%)	42 (3,51%)
*ST8*	103 (1,30%)	54 (0,81%)	49 (4,09%)
*ST9*	72 (0,91%)	72 (1,08%)	0 (0,00%)
*ST10*	124 (1,57%)	0 (0,00%)	114 (9,52%)
*ST12*	3 (0,04%)	3 (0,05%)	0 (0,00%)
*ST13*	1 (0,01%)	1 (0,02%)	0 (0,00%)
*ST14*	59 (0,75%)	0 (0,00%)	59 (4,92%)
*ST16*	9 (0,11%)	9 (0,14%)	0 (0,00%)
*ST17*	5 (0,06%)	0 (0,00%)	5 (0,42%)
*ST21*	31 (0,39%)	0 (0,00%)	31 (2,59%)
*ST23*	30 (0,38%)	0 (0,00%)	30 (2,50%)
*ST24*	71 (0,90%)	0 (0,00%)	71 (5,93%)
*ST25*	26 (0,33%)	0 (0,00%)	26 (2,17%)
*ST26*	27 (0,34%)	0 (0,00%)	27 (2,25%)
*ST27*	5 (0,06%)	0 (0,00%)	5 (0,42%)
*ST28*	5 (0,06%)	0 (0,00%)	5 (0,42%)
*ST29*	2 (0,03%)	0 (0,00%)	2 (0,17%)
*ST30*	11 (0,14%)	0 (0,00%)	11 (0,92%)
*ST31*	19 (0,24%)	0 (0,00%)	19 (1,59%)
*ST32*	2 (0,03%)	0 (0,00%)	2 (0,17%)
*Mixed*	42 (0,53%)	42 (0,63%)	0 (0,00%)
*Novel*	21 (0,27%)	20 (0,30%)	1 (0,08%)
*Not subtyped*	3738 (47,33%)	3267 (49,05%)	471 (39,32%)
*Total of samples*	7898 (100%)	6660 (100%)	1198 (100%)

### 3.2 Distribution of *Blastocystis* subtypes by country

**Figure 2:**
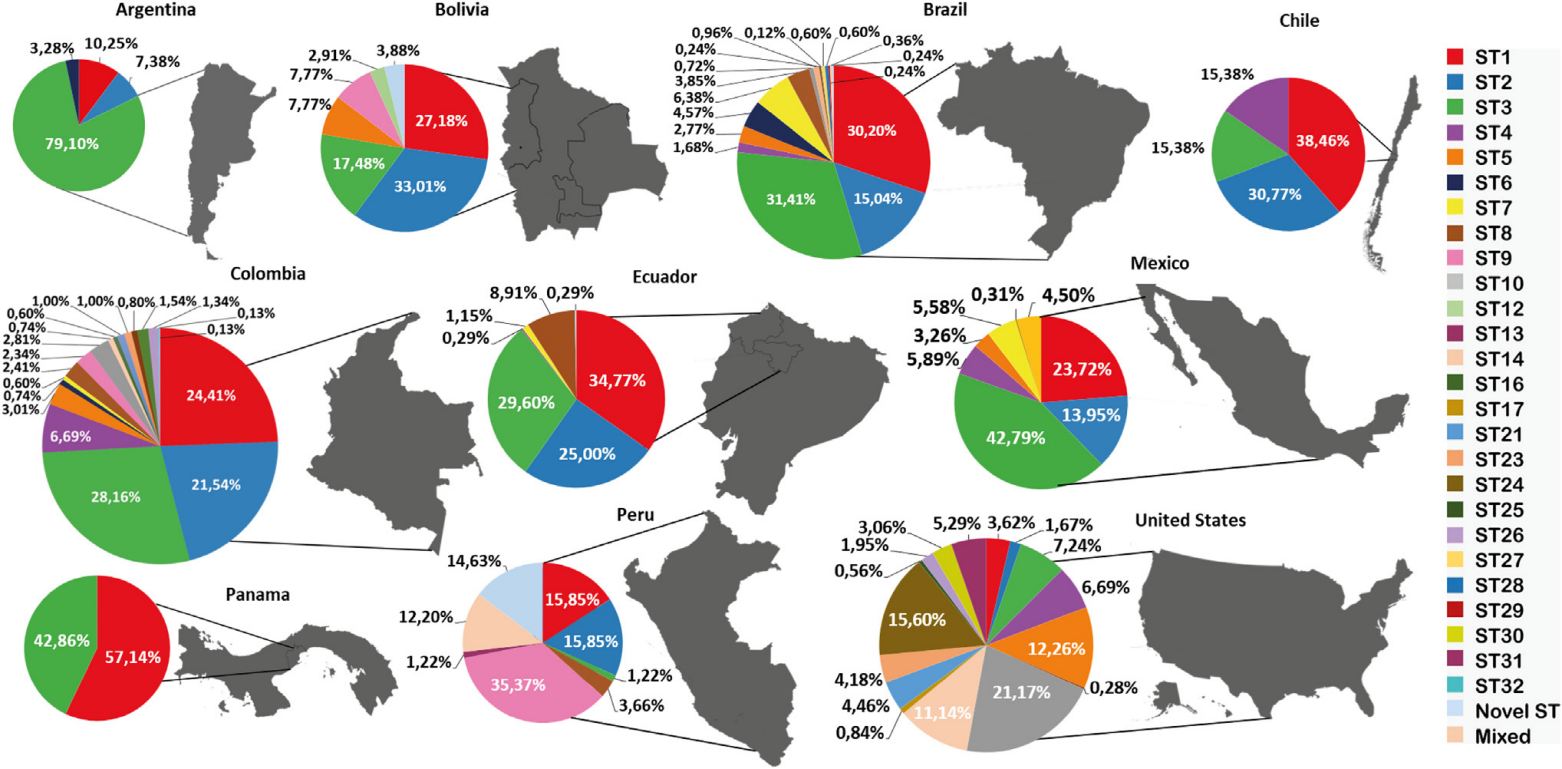
Distribution of *Blastocystis* subtypes by country. The reported positive samples from each country in the Americas were considered, and a distinct color separated each subtype. It is shown the percentages of STs by country.

*Blastocystis* subtypes distribution throughout the American continent is supported by the reports in recent decades that have shown the presence of the subtypes in ten (10) different countries (Argentina, Bolivia, Brazil, Chile, Colombia, Ecuador, Mexico, Panamá, Perú, and United States). In Figure 1, Honduras, Nicaragua, and Venezuela have identified the presence of the protozoan but have not been subtyped. The country with the greatest variety of subtypes was Colombia, reporting 18 different subtypes so far [9, 35, 43, 76, 77, 78, 79, 80, 81, 82, 83, 84], followed by the United States, reporting 16 subtypes [10, 48, 105, 106, 107, 108, 109, 110, 111, 112, 113], Brazil reporting 15 subtypes [39, 47, 55, 56, 57, 58, 59, 60, 61, 63, 64, 65, 66, 67, 68, 69, 70, 76, 116]. Other countries, such as Mexico, have reported seven subtypes [40, 49, 90, 94, 95, 96]. Bolivia [47, 52, 53], Peru [47, 50, 100, 101, 102, 103, 104] and, Ecuador six subtypes [47, 87, 88], Argentina and Chile four subtypes [24, 47, 74, 75], the country with the least variety of subtypes is Panama reporting two subtypes [99] (Figure 2).

**Figure 3:**
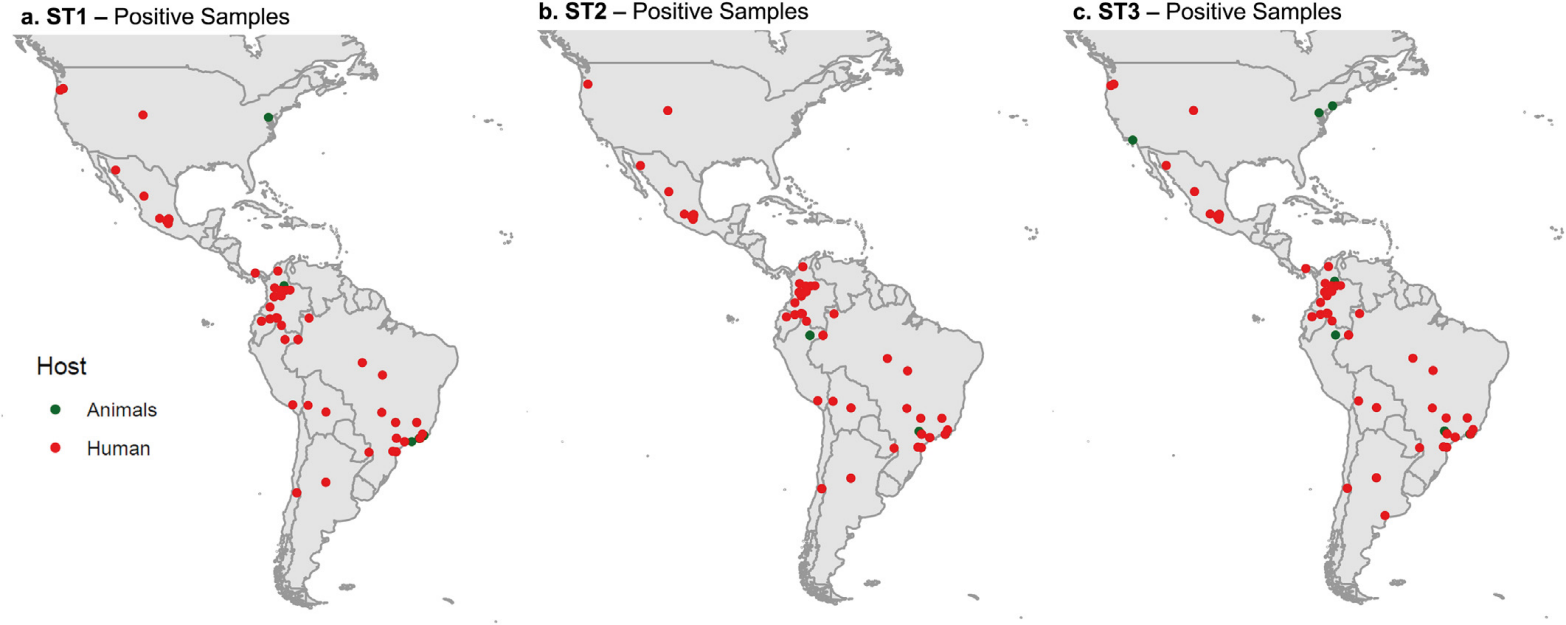
a. Distribution by country of the samples positive for ST1 in humans and other animals. b. for ST2 in humans and other animals. c. ST3 in humans and other animals.

ST1 is the most widely distributed, present in the 10 countries with positive human samples for *Blastocystis* in the Americas [9, 24, 31, 33, 40, 43, 47, 48, 50, 53, 55, 56, 57, 58, 59, 60, 61, 62, 63, 64, 66, 68, 69, 70, 74, 75, 76, 78, 79, 81, 83, 84, 87, 90, 91, 92, 93, 99, 106, 107, 108, 111, 112, 113, 116]. Analysis from the tests proved that Chile and Ecuador correlated with ST1 (*p =* 0.000107, *p =* 2.00E-06) correspondingly. Followed by ST2 [9, 24, 31, 40, 47, 52, 53, 55, 56, 57, 58, 59, 60, 62, 63, 64, 66, 67, 68, 69, 70, 74, 75, 77, 79, 83, 85, 87, 90, 91, 108, 112, 113, 116] and ST3 which have been reported in 9 countries except for Panama and Peru respectively [9, 10, 24, 40, 47, 52, 55, 56, 57, 58, 60, 62, 63, 64, 66, 68, 69, 70, 75, 81, 87, 90, 91, 93, 107, 108, 113, 116]. We then constructed georeferenced maps with specific locations from the positive samples from the most frequent subtypes (Figure 3). It should be noted that Figure 3a shows the distribution of ST1-positive samples from human hosts (red dots) and animal hosts (green dots). Figure 3b represents ST2 positive samples from humans (red dots) and animals (green dots). Finally, Figure 3c exhibits human (red dots) and animal (green dots) positive samples from subtype 3 (ST3).

Although ST1 is widely distributed in human samples throughout the continent, positive samples from animal hosts were limited to the following countries: Brazil [56, 57, 58, 69], Colombia [43, 77], México [97], and the United States [48, 111]. The most common animal host were mammals such as pigs, dogs, cats, and white-tailed deer. Interestingly, ST2-positive samples from animal hosts were found only in primates from Brazil [58, 69] and Perú [101]. On the other hand, ST3 positive samples distribution from an animal host was also limited across Brazil [57, 58, 69], Colombia [43], Perú [101], and the United States [10, 48, 111], being its significant representatives' of mammals and primates.

Other subtypes have limited distribution. Some subtypes have been described in only one country. ST12 has been described in human host samples only in Bolivia [47]; data analysis has shown a statistically significant association between Bolivia and this subtype (*p =* 1.40E-05). Likewise, ST13 has only been described in human hosts from Peru [47]. ST16 in Colombian human hosts [31], and recently in this country, a new subtype (ST32) was identified in cows and goats [43]. Some unique subtypes, ST27, ST28, and ST29, were identified in wild bird species [39, 71]. Furthermore, the latest studies on white-tailed deer in the United States demonstrated the presence of new subtypes, such as ST30 and ST31 [48]. Even though ST7 and ST8 had been reported in different countries, we found that they are correlated with Brazil (*p =* 0.002955; *p =* 6.00E-06).

It is important to recognize that samples in all the countries mentioned are reported in human hosts but not in all animal host samples described. Some countries such as Brazil [39, 56, 57, 58, 64, 65, 69, 71], Colombia [43, 77], Ecuador [87], Mexico [94], Peru [101, 104], and United States [10, 48, 105, 109, 110, 111], have demonstrated the presence of this protozoan in animals. Hence, the number of samples between humans and animals varies.

### 3.3 *Blastocystis* STs by animal hosts

The animal hosts in which *Blastocystis* positive samples were recorded include poultry [39, 58, 65, 71, 72], an heterogenic group of primates [58, 69, 87, 101, 104, 117], other mammals [48, 58, 69, 72, 94] including domestic animals [10, 43, 56, 57, 72, 73, 77, 105, 109, 110]. The wide variety of species reported as animal hosts is shown in Table 2 (Table 2). ST5 showed a correlation with *Gallus gallus, Leopardus tigrinus,* and *Mazama gouazoubira* (*p =* 0.017635; *p =* 0.017635; *p =* 0.017635). ST6 with *Phasianus colchicus* (Pheasant) (*p =* 0.00266), ST7 with *Anas platyrhynchos* (Wild Duck) and *Anser cygnoides* (Swan Goose) (*p =* 7.00E-06). ST8 with *Alouatta caraya, Ateles* sp., *Dasypus septemcinctus, Nectomys squamipes* (*p =* 0.006597), ST24 with *Struthio camelus* (Ostrich) (*p =* 0.001388) and ST32 with Goats (*p =* 0.00061).

**Table 2:** Animal Host species in *Blastocystis* positive samples.

	Host species	References
Poultry		
	*Anas* spp	[58, 71]
	*Anser* spp (Goose)	[58, 71, 72]
	*Gallus gallus*	[58]
	Chicken	[39, 65, 72]
	*Pavo cristatus (Indian Peafowl)*	[71]
	*Struthio camelus (*Ostrich)	[71]
	*Agapornis nigrigenis (*Black-cheeked Lovebird*)*	[71]
	*Numida meleagris* (Helmeted Guineafowl)	[71, 72]
	*Cairina moschata momelanotus* (Muscovy Duck)	[71]
	*Coturnix coturnix* (Quail)	[71]
	*Phasianus colchicus* (Pheasant)	[71]
Primates		
	*Ateles* sp	[58, 69, 101]
	*Alouatta* spp	[58, 69, 87]
	*Aotus* sp	[58, 104]
	*Callithrix* spp	[69, 117]
	*Callicebus lucifer*	[101]
	*Cebus* spp	[69, 117]
	*Lagothrix lagotricha*	[58, 69, 101]
	*Leontopithecus chrysomelas*	[69]
	*Macaca* spp	[58]
	*Mandrillus sphinx*	[69]
	*Pan troglodytes*	[58]
	*Papio* spp	[58, 69]
	*Pithecia monachus*	[101]
Mammals		
	*Akodon* spp	[58]
	*Cuniculus paca*	[72]
	*Dasypus* spp	[58, 72]
	*Dicotyles tajacu*	[72]
	*Herpailurus yaguarondi*	[69]
	*Leopardus* spp	[69]
	*Panthera onca*	[69]
	*Nasua nasua*	[69]
	*Procyon cancrivorus*	[69]
	*Blastocerus dichotomus*	[69]
	*Mazama gouazoubira*	[69]
	*Pecari tajacu*	[69]
	*Myrmecophaga tridactyla*	[69]
	*Hydrochoerus hydrochaeris*	[69, 72]
	*Didelphis* spp	[58, 69]
	*Akodon* spp	[58]
	*Rattus rattus*	[58]
	*Metachirus nudicautatus*	[58]
	*Nectomys squamipes*	[58]
	*Dasypus septemcinctus*	[58]
	*Philander opossum*	[94]
	*Sturnira lilium*	[94]
	*Heteromys spp*	[94]
	*Sus scrofa*	[58, 72]
	White-tailed deer	[48]
Domestic Animals		
	Cattle	[10, 43, 57, 72, 73, 109, 110]
	Pig	[43, 56, 57, 72, 105, 109]
	Minipig	[43]
	Sheep	[43, 72, 73]
	Dog	[43, 56, 64, 73, 77, 111]
	Horse	[43, 72]
	Llama	[43]
	Rabbit	[43]
	Cat	[73, 111]
	Goat	[43, 72]
	Donkey	[72]
Other Animals		
	*Chelonoidis* sp	[58]
	*Periplaneta americana*	[58]
	*Crassostrea virginica*	[97]

The host that exhibited most correlations was cattle, our study demonstrated a statistically significant association among ST14 (*p =* 0.004407), ST17 (*p =* 0.040239), ST21 (*p =* 0.004817) and ST23 (*p =* 0.023683).

### *3.4* *Blastocystis* Alleles by ST

**Figure 4:**
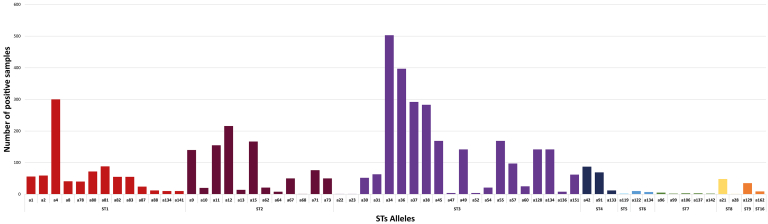
*Blastocystis* 18S Alleles distribution based on the positive samples for each subtype in human and animal hosts.

Considering the information obtained, sixty-two different alleles have been reported in *Blastocystis* subtypes in the Americas. It was found that the most frequent allele in animal and human hosts was ST3 (a34), present in 503 positive samples, followed by a36, reported in 397 positive samples from ST3, and a4 described in 300 positive samples from ST1. The alleles reported in each subtype are shown in Table S2 and below in Figure 4. Despite its wide distribution throughout the Americas, allele distribution is limited to countries such as Argentina, Brazil, Colombia, and the United States [9, 24, 31, 47, 56, 59, 62, 64, 67, 68, 69, 70, 76, 77, 83, 101, 112] (Figure 4; Table S2).

As described above, most allelic discrimination is reported in humans. However, some alleles have been identified in a few animal hosts. ST1 a4 is described in *Nasua nasua* and dogs; a1 in *Hydrochoerus hydrochaeris* [69, 77]. ST2 alleles are found in primates, a11 *Ateles belzebuth* and a12 in *Lagothrix lagotricha* and *Callicebus lucifer* [101], a15 in *Alouatta seniculus*, and a68 in *Ateles belzebuth*. Alleles exhibited in ST3 from animal hosts mainly found in primates were as follows: a34 in *Alouatta seniculus*, a22 in *Mandrillus sphinx*, a23 in *Papio hamadryas*, and a31 in *Papio Papio*. ST5 allele a119 was reported in Brazilian mammals such as *Leopardus tigrinus* and *Mazama gouazoubira.* ST8 a21 was described in primates *Alouatta caraya*, *Alouatta seniculus, Ateles fusciceps, Lagothrix lagotricha*, and a28 identified in *Hydrochoerus hydrochaeris* [69]*.*

The alleles information was obtained from those studies, which included in their methodology allelic typing (n = 16)—summarizing the information from those studies, which performed allele identification from both human and animal *Blastocystis* positive samples, in Table S2. Not every allele had a representative amount, and some alleles had less than 10% representation in each subtype. There are more samples without allelic identification in most STs, mainly in recent studies.

## 4 Discussion

*Blastocystis* is the most frequent enteric protozoan found in animal and human hosts worldwide [12, 118]. Our findings suggest that the number of countries reporting *Blastocystis*-positive samples and its subtypes has increased in recent years, providing valuable information about new hosts, transmission patterns, and ecological aspects and helping to clarify genetic and evolutionary changes among subtypes.

Brazil, Colombia, Mexico, and the United States have been highlighted as the major representatives in terms of *Blastocystis* reports in the Americas [31, 39, 43, 48, 57, 67, 68, 69, 70, 72, 80, 81, 82, 83, 84, 85, 94, 95, 96, 112, 117, 119, 120]. Our study demonstrated that Colombia provides the largest number of *Blastocystis*-positive samples (1495), mainly from ST1, ST2, ST3, and ST4, and the country with the highest variability of reported subtypes [9, 31, 43, 47, 76, 77, 78, 79, 81, 83, 84]. Colombia is considered a heterogeneous country regarding geography, climate, ecosystems, and biodiversity. Its biogeographical regions have their features, including variations in socioeconomic conditions, health care, waste collection, sewage treatment, and cultural and behavioral factors. These considerations could be linked to the high frequency of intestinal pathogens due to factors that may favor their transmission [83, 121]. According to data from the Institute of Hydrology, Meteorology and Environmental Studies (IDEAM - Instituto de Hidrología, Meteorología y Estudios Ambientales), population growth has driven an expansion of economic activities, which influences the reduction in the availability of resources in the country [122]—leading to a disturbance in the ecological niches where some pathogens may be found, possibly promoting their propagation, and affecting their circulation.

Brazil, Mexico, Argentina, and Ecuador [24, 33, 40, 47, 51, 55, 56, 57, 58, 59, 60, 62, 64, 66, 69, 87, 88, 90, 91, 92, 93, 95, 96, 116, 123] have also contributed a large number of positive samples for ST3, ST1 and ST2 exceeding one hundred samples per country. Interestingly, we observed a correlation between human hosts and the subtypes mentioned. This suggests a particular role of these STs in the Americas, and their preponderant distribution might be related to particular regional features. More studies are needed to identify the biological properties and their implications for virulence and disease outcomes.

Previous studies in Europe, Asia, and Oceania highlighted the genetic diversity of this protozoan. Numerous reports in European countries have shown that ST4 is the most common subtype in human samples [36, 44, 124, 125]. However, it is also reported a high prevalence of ST1, ST2, and ST3 in Europe, Australia, and Southeastern Asian countries [44, 126, 127]. There can be contrasting realities within the countries that conform to each continent. Nemati et al. published an overview of *Blastocystis* subtypes reported in Asia and Australia, indicating that ST1 and ST3 were the most prevalent subtypes from human samples in Asian countries [128]; in southern countries such as Thailand, these subtypes were found in human samples as well [44].

Additionally, other subtypes have been described in this country (ST2, ST10, ST11, ST13, ST14) [128]. Unlike European countries, the actual source of ST4 is unclear in the southern Asian region; it is more common in East Asian countries and zoonotic subtypes ST6 and ST7 [44, 129]. Based on these findings, the authors suggested that the transmission and distribution patterns may be affected by climate and geographic features, not only by socioeconomic conditions. As the tropical climate increases, the prevalence of this protozoan changes, which is reflected in the high frequency of some STs in eastern than western Asian countries [129]. The communities living close to their animals, belonging to a rural environment with a single water source, could explain the presence of previously undescribed subtypes in humans (ST23 and ST10), suggesting the importance of identifying the different routes of parasite transmission [44].

These differences between continents highlight the importance of further investigating the molecular distribution of *Blastocystis* to explain if ST3 is associated with transmission patterns between humans and identify the spreading mechanism of ST1-ST4 in non-human hosts. Also, it is essential to add new phenomena related to the distribution of *Blastocystis* and its subtypes, understanding these factors as climate, socioeconomic conditions, hydrographic distribution, water management, and treatment, among others that may be associated with the prevalence in different geographical locations.

Even though subtypes 1 to 9 are primarily described in humans, the information obtained from this review revealed that in smaller percentages, ST1-ST8 were found in animal hosts, including different species [10, 43, 48, 56, 57, 58, 69, 77, 94, 97, 101, 105, 109, 111]. Some STs showed a strong correlation between specific animal hosts, particularly ST5-ST8 (Table S3). Our analysis showed several associations between the same subtype with different hosts, as well as it was demonstrated that the same host could have significant associations with multiple subtypes, one of them being cattle, which showed correlations with more than one subtype (ST14, ST17, ST21, and ST23). Our results indicate that livestock could be a risk factor for *Blastocystis* transmission, which reinforces the idea of possible interactions through contact between animals from the same and different species, humans, and their environment.

Furthermore, ST12, ST13, and ST16 were reported in human hosts instead of animal hosts from Bolivia, Peru, and Colombia [31, 47]. The first two subtypes reported by Ramirez et al., in 2016 showed contrasting results, ST12 and Bolivia were significantly correlated, but ST13 and Peru were not correlated. ST16 was reported by Osorio-Pulgarín et al. in 2021, where there was no correlation with Colombia either; there have been no new reports of these subtypes in humans or animals, which leads to thinking about *Blastocystis* zoonotic potential. This indicates that there might be a relationship or contact between animal hosts and human hosts either by domestication, farming, or zootechnic production, which has allowed the colonization of the different subtypes of human potential and its spread to animal hosts. The hypothesis of multiple transmission mechanisms, including anthropo-zoonotic relationships, arises in those subtypes defined in human hosts as ST3. Not to mention other transmission routes such as human-to-human, animal-to-human, and presumably environment-to-host. However, the approach of defining particular transmission routes for each subtype should be studied and include environmental samples to unveil the life cycle of *Blastocystis*.

Additionally, new subtypes have been described in the Americas, and most of them have been identified in animal hosts. Novel subtypes such as ST24, ST25, and ST27-ST29 were not previously reported in some countries such as Colombia and Brazil [39, 71]. New subtypes in the United States, such as ST30 and ST31, were first detected in white-tailed deer, including ST21 and ST23-ST26 [48]. Higuera et al. reported subtypes ST21, ST23-ST26 in Colombian sheep, goats, horses, llamas, and cattle. Furthermore, they detected a previously reported new subtype, ST32, in goats and cattle [43]. These recent novel subtypes demonstrate an innovative approach to estimating *Blastocystis* genetic diversity and emerging subtypes. This suggests that using NGS (Next Generation Sequencing) strategies will facilitate our understanding of the molecular epidemiology of *Blastocystis* and the implications of mixed STs within individuals.

The distribution of *Blastocystis* in the Americas has exposed an increase in the number of reports in the last few years and a growing interest in identifying subtypes. This is supported by new methodological subtyping techniques such as next-generation sequencing and criteria to establish the nomenclature of novel subtypes [39]. Complete sequencing of the 18S-rRNA gene has opened the door to many possibilities for identifying subtypes. It could bring a closer look to elucidate the geographical and molecular distribution of the protozoan in a more precise and complete way, hence the importance of having more countries and researchers using these methods.

This work shows that the use of NGS has decreased the reports of “Novel ST” terminology in the last two years, increasing the identification of numerical classification of subtypes, including the description of new subtypes in North and South America [39, 43, 48]. Since the information on these novel subtypes is still limited to certain countries, the application of these methods has been reported mainly in Brazil, Colombia, and the United States, and the reports before 2019 use different molecular techniques. It should be considered to continue sequencing the complete gene (18S) to provide genuinely novel STs and determine the genetic differences between the detected STs throughout the continent. Lastly, as NGS continues to be employed by researchers across the Americas (sequencing capacity increased due to the worldwide efforts deployed for genomic surveillance during the COVID-19 pandemic), novel targets should be developed, or even Whole Genome Sequencing typing strategies should be in place for the better comprehension of *Blastocystis* genetic diversity.

Thirty-five nations integrate the American continent, and *Blastocystis*-positive samples have been reported in thirteen countries. Despite the interest in the molecular identification of *Blastocystis*, more investigation is needed to explain the unknown data, to have a complete molecular distribution description of this protozoan, and encourage those twenty-two countries that have not yet reported or subtyped it. The previous statement could indicate that most countries of the continent are developing countries; access to new technology, molecular tools, and reagents can be expensive. Therefore, it can be considered a limitation in achieving molecular diagnosis and robust analysis in parasite research. Understandably, most of the reports in this study are performed by microscopy identification; those with access to advanced tools such as Next Generation platforms are limited.

## 5 Conclusions

In the last two decades, attempts have been made to demonstrate *Blastocystis* genetic variability and the differences between subtypes. Likewise, its prevalence in the world is also highly variable, drastically impacting its geographic distribution worldwide. This molecular and geographic variety has been shown in the American continent. Countries such as Colombia, Brazil, Mexico, Peru, and the United States have promoted the publication of new studies that support the update of valuable epidemiological data. However, the information obtained allowed us to update the existing subtypes but is insufficient to determine the current subtypes circulating in the Americas as a whole; this is only a slight approximation.

Additionally, we remark to consider in further studies environmental factors such as biodiversity, climatology, hydrographic, and sociodemographic, among others, which could explain the emergence of novel subtypes or changes in its distribution. We encourage countries that have already demonstrated the protozoan presence to conduct subtyping studies. Equally, we suggest applying new subtyping methods, such as the complete sequencing of the 18S-rRNA gene, to adequately detect new and currently circulating subtypes, maintaining the criteria to avoid typing errors or wrong molecular distribution. Each researcher in the *Blastocystis* community is responsible for maintaining an adequate *Blastocystis* nomenclature and promoting valuable information for the population, generating awareness of the existence and prompt identification of potentially pathogenic microorganisms such as *Blastocystis*. Finally, we call upon scientists to continue innovating methodologies and maintain an interest in identifying this parasite, whose importance lies in its high prevalence.

## Declarations

### Author contribution statement

Paula Jiménez: conceived and designed the study; performed the study; analyzed and interpreted the data; wrote the paper.

Marina Muñoz: conceived and designed the study; analyzed and interpreted the data; wrote the paper.

Juan David Ramírez: conceived and designed the study; performed the study; analyzed and interpreted the data; wrote the paper.

### Funding statement

This research did not receive any specific grant from funding agencies in the public, commercial, or not-for-profit sectors.

### Data availability statement

Data included in article/supp. material/referenced in article.

### Declaration of interest’s statement

The authors declare no conflict of interest.

### Additional information

The following supporting information can be downloaded at CIMBIUR Github https://github.com/gimur/Blastocystis_STs_P.

## Appendix A Supplementary data

Table S1. Blastocystis Database Table S1. Blastocystis Database

Table S2. Distribution of Blastocystis 18S alleles by STs Table S2. Distribution of Blastocystis 18S alleles by STs

Table S3. Correlation STs -Country-Hosts Table S3. Correlation STs -Country-Hosts
